# Shared-Scale Predictive Benchmarking of an Acoustic-Radiation-Force Model for Frequency-Dependent Retinal Ganglion Cell Responses

**DOI:** 10.3390/bioengineering13090979

**Published:** 2026-08-26

**Authors:** Bingao Zhang, Shengyong Xu

**Affiliations:** The Key Laboratory for the Physics and Chemistry of Nanodevices, Institute of Physical Electronics, Department of Electronics, Peking University, Beijing 100871, China; zhangbingao@pku.edu.cn

**Keywords:** ultrasonic neurostimulation, acoustic radiation force, Hodgkin–Huxley model, retinal ganglion cells, phenomenological inhibitory current, biophysical simulation, retinal prosthetics

## Abstract

Retinal responses to ultrasound depend on carrier frequency, but whether a constrained tissue-to-neuron model can predict this dependence on a common published response scale remains unclear. We developed an acoustic-radiation-force (ARF) modelling workflow that couples an effective tissue-scale stress proxy to a stochastic Hodgkin–Huxley retinal ganglion cell population. One non-negative affine observation model, shared across frequencies, linked raw simulated spike counts to the published three-frequency ex vivo RGC response scale without frequency-specific rescaling. In a nested leave-one-frequency-out model-selection assessment, the workflow achieved an overall $Q^{2}$ of 0.915 and an RMSE of 0.0752 across 25 held-out observations. Mean-response transfer remained strong across frequencies, although probabilistic calibration was weakest at 1.9 MHz. An in-sample fixed-map sensitivity analysis using moment-matched log-normal, Gamma, and Weibull thresholds yielded overall RMSEs of 0.0434–0.0461 and preserved positive overall K-ablation NLPD differences of 0.219–0.238, with the effect concentrated at 43 MHz. These results show that shared-scale modelling captures the main cross-frequency response structure and that a high-threshold inhibitory functional component improves the description of high-intensity rolloff. Within the published three-frequency dataset, the workflow provides a reproducible benchmark for targeted comparisons of retinal ultrasound mechanisms.

## 1. Introduction

Restoring vision in outer retinal degenerative diseases such as retinitis pigmentosa (RP) and age-related macular degeneration (AMD) remains a major challenge in neural engineering. In these disorders, photoreceptors degenerate while a large fraction of the inner retinal circuitry, including bipolar cells and retinal ganglion cells (RGCs), persists for extended periods. This residual circuitry motivates prosthetic strategies that bypass the lost photoreceptor layer and directly engage surviving neurons. Electronic retinal prostheses have established clinical feasibility, exemplified by the Argus II epiretinal implant [1] and the PRIMA subretinal photovoltaic array [2], but their clinical use remains constrained by invasive implantation and by electrode–tissue interface and spatial-resolution trade-offs [3].

Ultrasound stimulation is being explored as a non-contact candidate strategy for retinal prosthetics because acoustic energy can be delivered through the anterior segment without placing a stimulating array on the retina. Early feasibility studies proposed multifocal ultrasonic stimulation as a route to patterned, non-contact retinal activation [4], and subsequent in vivo and ex vivo studies reported stimulus-evoked neural responses under several retinal stimulation configurations [5,6,7]. In parallel, device-oriented simulations have optimised transducer geometry and focal fields for patterned stimulation [8,9]. These hardware studies are important for prosthesis development, but they do not themselves explain how a given acoustic field is transformed into a frequency-dependent RGC firing response.

Three classes of mechanism have been discussed in ultrasound neuromodulation: thermal effects, cavitation-related effects, and acoustic radiation force (ARF) [10,11,12,13]. For the MHz range retinal protocol studied by Menz et al., the reported heating estimates did not directly account for the frequency-dependent response pattern, although thermal contributions cannot be excluded without protocol-specific measurements [14]. Cavitation-based models, including the neuronal intramembrane-cavitation excitation (NICE) framework [15,16] and its cycle-averaged SONIC reduction [17], provide a concrete electromechanical account of neural excitation developed primarily for lower carrier-frequency regimes (≤500 kHz). The retinal stimulation context studied here differs materially: the tissue is thin (≈150 μm), mechanically constrained, fluid-bathed, and stimulated at 1.9–43 MHz. These conditions do not exclude cavitation, but low-frequency NICE/SONIC assumptions cannot be directly extrapolated without physical justification compatible with MHz carrier frequencies, local mechanical constraints, and tissue geometry.

ARF is a plausible candidate mechanism because it scales with acoustic intensity and tissue absorption, and the attenuation coefficient of retinal tissue increases with carrier frequency [14,18]. Under an ARF-dominant hypothesis and otherwise comparable exposure conditions, increasing the carrier frequency raises the effective radiation-force input. Menz et al. [14] tested this frequency dependence directly across 1.9, 15, and 43 MHz. Retinal activation thresholds decreased with increasing frequency. This pattern is consistent with the ARF hypothesis but does not by itself exclude thermal, cavitation-related, or mixed mechanisms. Their work supports ARF as an important physical candidate for the retinal response, but the cellular pathway that links ARF-induced tissue displacement to a frequency-dependent population firing output remains unresolved.

A plausible modelling route is to test phenomenological current surrogates rather than assign a molecular channel identity. The TREK/TRAAK sub-family of two-pore domain K^+^ (K2P) channels is gated by lateral bilayer tension through a force-from-lipids mechanism [19,20]. These channels provide a biophysical reference for an outward, hyperpolarising current because their tension-gating properties have been quantitatively characterised [21]. Ultrasound can modulate ion-channel currents, including mechanosensitive K^+^ currents, in reduced cellular preparations [22]. K2P-family channels are also expressed in mouse retina and ganglion-cell-layer regions [23], and TREK/TRAAK expression is elevated in rd1 degenerating retina [24]. Together, these observations motivate an outward K^+^ current-like phenomenological alternative. In this study, TREK/TRAAK and related mechanosensitive K^+^ channels provide biophysical background for this alternative, not molecular targets identified by the model. The branch is used to assess whether an inhibitory membrane-current form helps describe high-intensity response rolloff; its frequency-dependent contribution is evaluated by ablation analysis.

This work draws on three lines of prior computational research. Device-oriented retinal-ultrasound simulations have focused on transducer geometry, focal-field optimisation, and the feasibility of patterned stimulation [4,7,8,9], without a mechanistic population-level model of channel-mediated transduction. General ARF analyses have examined stress generation in neural tissue [25] but were not formulated to explain high-frequency, thin-retina dose–response behaviour. The NICE/SONIC framework [15,17] provides a mechanistic electromechanical theory for cavitation-driven neuromodulation at low carrier frequencies but does not directly address the MHz retinal context described here. What remains missing is a constrained and reproducible workflow for asking whether one physical-to-neuronal chain can describe frequency-dependent retinal response curves on a common scale. Such a workflow must link acoustic exposure, radiation-force-driven tissue deformation, membrane excitability, and population-level activation while keeping mechanism comparisons within the same observation model. The retinal preparation studied by Menz et al. [14] provides a useful benchmark because it reports activation across widely separated ultrasound frequencies and includes a separate tissue-displacement dataset. That displacement dataset supplies descriptive mechanical context without calibrating the HH model.

We address this gap by testing an ARF-centred workflow under explicit calibration constraints. The workflow couples a local travelling-wave ARF approximation [14,18], a one-dimensional continuum-mechanics stress model, and a Hodgkin–Huxley population model [26]. We link raw simulated spike counts to the published response scale with one observation model shared by all three frequencies. This common mapping provides a consistent statistical bridge between non-equivalent simulated and experimental endpoints without separate within-frequency normalisation. A nested leave-one-frequency-out model-selection assessment tests held-out mean-response transfer without frequency-specific response scaling. Fixed-observation-map ablations then assess the descriptive contribution of alternative transduction mechanisms. Together, these elements provide a constrained and reproducible route from acoustic exposure to held-out population responses and experimentally testable hypotheses.

## 2. Materials and Methods

### 2.1. Simulation Pipeline

**Figure 1:**
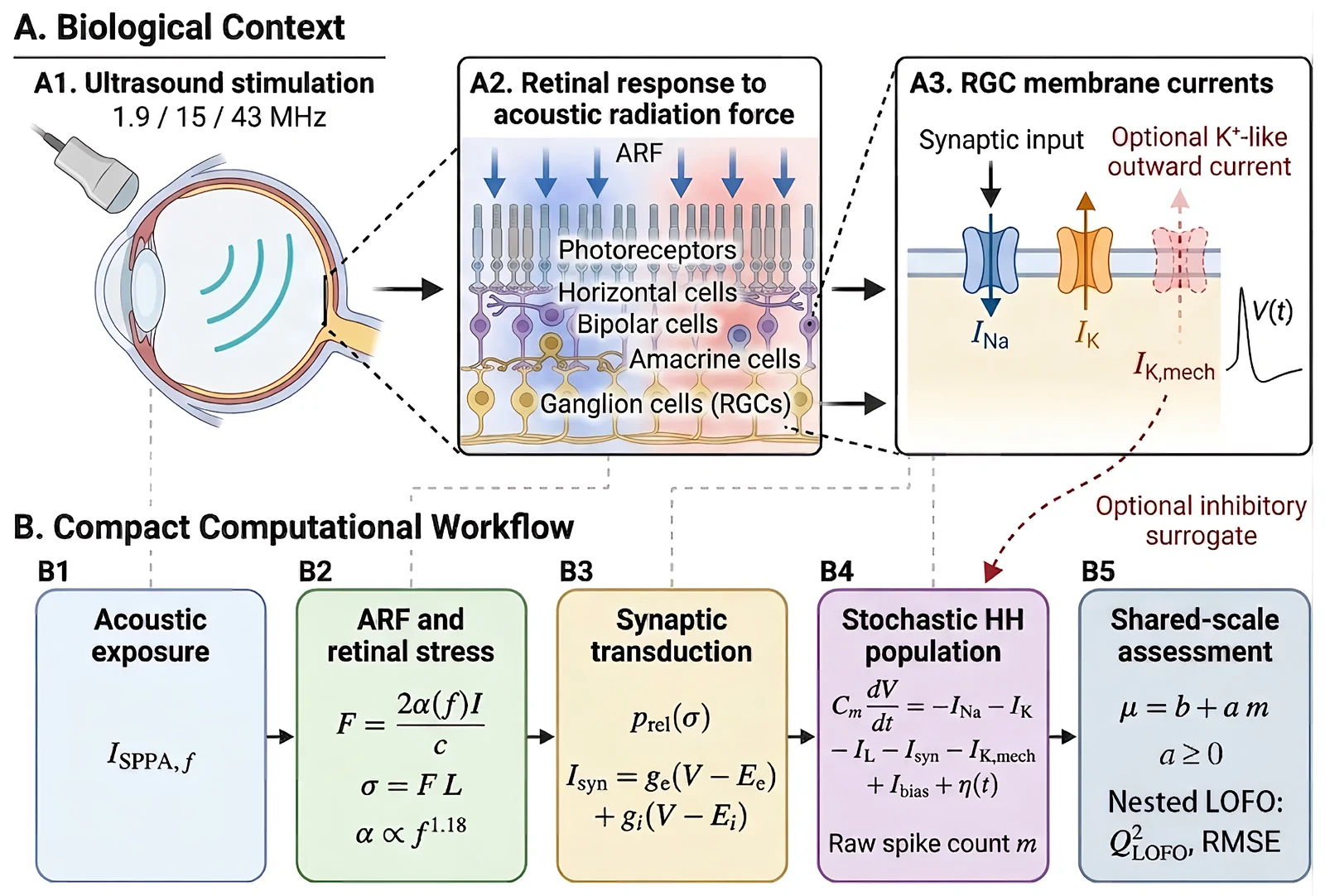
Biological context and computational workflow for frequency-dependent retinal ultrasound modelling. (**A**) (**A1**) Ultrasound at 1.9, 15, and 43 MHz is directed towards the retina. (**A2**) Acoustic radiation force (ARF) is shown schematically across the retinal layers. (**A3**) Synaptic input drives an HH excitable-membrane surrogate for the RGC population, with voltage-gated Na^+^ and K^+^ currents. The dashed red pathway denotes an optional phenomenological K-like outward current, $I_{K,\mathrm{mech}}$, without assigning a molecular channel. (**B**) (**B1**–**B4**) Acoustic exposure ($I_{\mathrm{SPPA}}$ and *f*) is mapped to a local travelling-wave ARF proxy, an effective retinal-stress proxy, synaptic input, and raw spike count *m* from a stochastic HH population. (**B5**) *m* is mapped to the published response scale with one non-negative affine observation model shared across frequencies, and evaluates held-out mean responses by nested leave-one-frequency-out assessment. The workflow uses no frequency-specific response rescaling.

The physical-to-electrophysiological chain in Figure 1 is organised into five stages: (i) convert spatial-peak pulse-average intensity ($I_{\mathrm{SPPA}}$, W/cm^2^) to a local travelling-wave ARF body-force proxy; (ii) convert this proxy to an effective bulk retinal stress; (iii) map stress to synaptic input across heterogeneous neuronal thresholds; (iv) integrate the HH equations and count spikes within the 100 ms stimulation window; and (v) map the raw population spike counts to the published response scale using one shared non-negative affine observation model. The benchmark pathway contains no direct excitatory mechanosensitive membrane current. K-OFF and the optional K-like inhibitory surrogate are candidate branches within the nested selection procedure described below. The published three-frequency RGC dose–response data reported by Menz et al. [14] provide the population benchmark.

### 2.2. Acoustic Radiation Force

For a plane ultrasound wave propagating in an attenuating medium, we represent the time-averaged ARF body-force density (N/m^3^) with a local one-dimensional travelling-wave approximation:

(1) $$F=\frac{2\alpha \left(f\right)\;I_{\mathrm{SPPA}}}{c}.$$

This quantity serves as an effective tissue-scale body-force surrogate rather than as a three-dimensional representation of focusing, standing-wave forces, cavitation, or local finite-element stress fields.

Here, α(f) is the amplitude attenuation coefficient (Np/m), $I_{\mathrm{SPPA}}$ is in W/m^2^, and c=1540 m/s is the longitudinal wave speed in retinal tissue (Supplementary Table S1; [14]). For benchmark consistency, we follow the parameterisation used by Menz et al. [14]: the 43 MHz retinal attenuation value comes from de Korte et al. [18], and the frequency exponent is the value adopted in the Menz ARF model:

(2) $$\alpha \left(f\right)=22.02\times {\left(\frac{f}{43}\right)}^{1.18}\;\left[\mathrm{dB}/\mathrm{cm}\right].$$

Conversion from dB/cm to Np/m uses the factor 1dB/cm=11.513Np/m. This yields α(43 MHz) =22.02 dB/cm (253.5 Np/m), α(15 MHz) ≈6.35 dB/cm (73.1 Np/m), and α(1.9 MHz) ≈0.556 dB/cm (6.40 Np/m). This frequency dependence (exponent 1.18, near-linear) is the main source of carrier-frequency variation in the ARF input used here.

### 2.3. Mechanical Stress Transduction

The ARF body-force density *F* (N/m^3^) is integrated over the retinal thickness to obtain the bulk compressive stress acting on the retinal layer:

(3) $$\sigma =F\cdot L_{\mathrm{retina}},$$

where $L_{\mathrm{retina}}=150\;\mu$m and σ is in Pa. When the transducer focal spot diameter (≳100μm; Menz et al. [14]) is comparable to or larger than $L_{\mathrm{retina}}$, integration through the retinal thickness provides a first-order bulk-stress approximation. The resulting proxy matches the spatial resolution supported by the acoustic measurements used for calibration. It does not reconstruct the three-dimensional ocular geometry, transducer focal field, layer-specific boundary conditions, reflections or standing waves, acoustic streaming, lateral and depth-dependent stress gradients, or local cellular strain. Throughout the model, σ therefore denotes an effective bulk-stress input to mechanotransduction rather than lateral membrane tension at a specific channel complex.

### 2.4. Independent Tissue Displacement–Population Activation Association

As an independent descriptive analysis, we fitted the published tissue displacement–population response data reported by Menz et al. [14]. The displacement was measured at a retinal tissue level and the neural response was obtained from a separate preparation; it is therefore neither a single-cell membrane-displacement measurement nor a paired cell-level calibration. With lateral retinal displacement *x* (μm) as the independent variable, the normalised population response r(x) was modelled as:

(4) $$r\left(x\right)=\frac{1}{1+e^{-k_{0}\left(x-x_{0}\right)}}\cdot e^{-x/x_{\mathrm{inh}}},$$

where $x_{0}$ is the half-activation displacement, $k_{0}$ controls sigmoid slope, and $x_{\mathrm{inh}}$ is the high-intensity inhibitory decay constant. Parameters were estimated by least squares from the published displacement–response data, with the fitted values reported in the Results. This fit summarises an empirical tissue displacement–population response association and remains separate from the three-frequency benchmark: its parameters are not passed to the stress-threshold, synaptic, or HH modules and do not calibrate an individual-RGC membrane-tension threshold.

### 2.5. Population Heterogeneity and Synaptic Mapping

Each neuron *i* (i=1,…,N) is assigned an independent mechanosensitive threshold $\sigma_{\mathrm{half},i}$ drawn from a log-normal distribution:

(5) $$\ln\sigma_{\mathrm{half},i}∼N\;\left(\mu_{\log},\;\sigma_{\log}^{2}\right).$$

The log-normal family was chosen as a parsimonious positive-valued model of population heterogeneity: many neural structural and functional quantities are right-skewed and approximately log-normal [27], whereas mechanosensitive activation thresholds can vary with membrane tension and cellular context [28]. Together, these observations motivate, but do not establish, a right-skewed distribution of effective mechanosensitivity across the modelled population. The shared log-normal profile was selected from a fixed 7×6 grid under the nested cross-frequency model-selection procedure described below. Because the Menz dataset does not contain cell-resolved mechanical thresholds [14], this distribution is a structural modelling assumption rather than an empirical estimate of RGC threshold statistics. The heterogeneity term represents effective variation in mechanosensitive thresholds and local microenvironments across the RGC population, not measured cell-by-cell channel expression. The population size depended on the analysis stage: candidate screening used N=15, whereas final full-data curves and downstream comparisons used N=60. Trial and seed settings are specified below.

Stress σ is mapped to single-neuron synaptic release probability via a logistic function:

(6) $$p_{\mathrm{rel},i}\left(\sigma \right)=\frac{1}{1+\exp\;\left(-\frac{\sigma -\sigma_{\mathrm{half},i}}{k_{s,i}}\right)}.$$

The slope is defined per neuron as $k_{s,i}=\sigma_{\mathrm{half},i}/2$; it is therefore relative to the sampled half-activation threshold rather than a fixed value in Pascals. This release probability is convolved separately with excitatory ($\tau_{e}=2.5$ ms) and inhibitory ($\tau_{i}=9.0$ ms) alpha kernels $h\left(t\right)=\left(t/\tau^{2}\right)e^{-t/\tau }$ to produce time-varying synaptic conductances:

(7) $$g_{e}\left(t\right)=a_{e}\cdot \left(p_{\mathrm{rel}}*h_{e}\right)\left(t\right),\;g_{i}\left(t\right)=a_{i}\cdot \left(p_{\mathrm{rel}}*h_{i}\right)\left(t\right).$$

The nested candidate library used

$$a_{e}\in \left\{0.28,0.35,0.42\right\},\;a_{i}\in \left\{0.112,0.14,0.168\right\}\;{\mathrm{mS}/\mathrm{cm}}^{2}.$$

One pair was selected from the training frequencies in each held-out fold. The net synaptic current injected into the HH model was:

(8) $$I_{\mathrm{syn}}\left(t\right)=g_{e}\left(t\right)\left(V-E_{e}\right)+g_{i}\left(t\right)\left(V-E_{i}\right),$$

with reversal potentials $E_{e}=0$ mV (excitatory) and $E_{i}=-72$ mV (inhibitory).

### 2.6. Threshold-Distribution Sensitivity Analysis

To test whether the final conclusions depended on the selected positive-valued threshold family, we compared the baseline log-normal distribution ($\mu_{\log}=-0.25$, $\sigma_{\log}=0.60$) with Gamma and Weibull alternatives matched to the same mean (0.9324) and coefficient of variation (0.6583). The resulting Gamma shape and scale were 2.3077 and 0.4040, and the Weibull shape and scale were 1.5513 and 1.0368, respectively. For each seed, common uniform quantiles were transformed through the three inverse cumulative distribution functions, with extreme quantiles clipped for numerical stability. We retained N=60, K=3, the 30 final random seeds, all synaptic, OU, and K-like parameters, and the selected affine observation mapping (a=0.0798, b=0.0462, τ=0.0278). No parameter or observation-scale refitting was allowed. We report overall and frequency-specific RMSE, NLPD, 95% prediction-interval coverage, and the fixed-map K-OFF minus K-ON NLPD difference; uncertainty summaries used 2000 paired seed-bootstrap resamples. Complete implementation and results are provided in the Supplementary Methods and Table S4.

### 2.7. Hodgkin–Huxley Neuron Model

Following established conductance-based retinal ganglion cell formulations [29], each of the *N* neurons obeys the standard Hodgkin–Huxley equations driven by the phenomenological synaptic current and, in K-ON runs, an optional inhibitory K^+^ current. This common HH module is used as an excitable-membrane surrogate; it does not imply that ON and OFF RGC subtypes share identical conductances, firing modes, or channel composition. Effective threshold heterogeneity can absorb only part of this unresolved diversity and does not replace an explicit retinal circuit model:

(9) $$C_{m}\frac{dV}{dt}=-I_{\mathrm{Na}}-I_{K}-I_{L}-I_{\mathrm{syn}}-I_{K,\mathrm{mech}}+I_{\mathrm{bias}}+\eta \left(t\right).$$

Here, $C_{m}$ is the membrane capacitance per unit area, *V* is membrane potential, and *t* is time. The voltage-gated currents are $I_{\mathrm{Na}}=g_{\mathrm{Na}}m^{3}h\left(V-E_{\mathrm{Na}}\right)$ for fast inward sodium and $I_{K}=g_{K}n^{4}\left(V-E_{K}\right)$ for delayed-rectifier potassium; $I_{L}=g_{L}\left(V-E_{L}\right)$ is the leak current. The *g* terms are maximal conductance densities, the *E* terms are reversal potentials, and *m*, *h*, and *n* are the sodium activation, sodium inactivation, and potassium activation variables, respectively. $I_{\mathrm{syn}}$ is the net synaptic current defined in Equation (8). $I_{K,\mathrm{mech}}$ is the optional stress-dependent outward K^+^ current defined in Equation (11) and is set to zero in K-OFF runs. $I_{\mathrm{bias}}=3.5\;\mu$A/cm^2^ is a tonic applied current representing baseline RGC excitability, and η(t) is a zero-mean stochastic applied current. Ionic and synaptic currents are defined as outward-positive and therefore enter Equation (9) with minus signs; positive $I_{\mathrm{bias}}$ and η(t) are depolarizing under this convention.

We represented η(t) by an Ornstein–Uhlenbeck (OU) process, a parsimonious stationary Gaussian coloured-noise model with a finite exponential correlation time that has been used to approximate temporally filtered background synaptic fluctuations in neuronal models [30,31]. Unlike temporally independent white noise, it preserves a millisecond-scale memory of recent input. The zero mean prevents the stochastic term from shifting the tonic drive already represented by $I_{\mathrm{bias}}$, while its variance controls trial-to-trial excitability fluctuations. The process is updated with the exact discrete transition

(10) $$\eta_{n+1}=a\eta_{n}+s_{\eta }\sqrt{1-a^{2}}\;\xi_{n},\;a=\exp\left(-\Delta t/\tau_{\eta }\right),\;\xi_{n}∼N\left(0,1\right),$$

where $\eta_{n}$ is the stochastic current at time step *n*, *a* is the one-step persistence coefficient, Δt is the integration step, $\tau_{\eta }$ is the correlation time, $\xi_{n}$ is an independent standard-normal innovation, and $s_{\eta }$ is the stationary standard deviation. Consequently, $s_{\eta }$ does not change when Δt changes. We set $\tau_{\eta }=5$ ms to represent millisecond-scale input correlations and selected one OU amplitude from $s_{\eta }\in \left\{0.30,0.40,0.50\right\}$ μA/cm^2^, shared across frequencies within each fold. This additive OU current is a reduced surrogate for unresolved background input, not an explicit conductance-based synaptic model. Its correlation time and amplitude are modelling assumptions, not measured RGC parameters. Classical HH gate kinetics and conductances [26] provide a tractable excitable-membrane surrogate rather than a cell-type-specific RGC reconstruction; the complete parameter set is listed in Supplementary Table S2.

Each simulation spans 150 ms: 20 ms baseline, a 100 ms continuous-wave pulse, and a 30 ms post-stimulus interval. Spikes are detected by V=0 mV upward crossings with a 1.2 ms absolute refractory period. The primary response is the mean spike count per neuron and trial within the stimulation window 20≤t<120 ms; baseline and post-stimulus spikes are not included. The corresponding firing rate in Hertz is the mean count divided by 0.1 s. The analysis uses the unnormalised mean-count output. A shared observation model maps this output to the published response scale. Numerical integration used Δt=0.02 ms. Representative timestep-refinement simulations and stationary-statistics checks of the OU input were used to assess numerical consistency; details are provided in the Supplementary Methods.

### 2.8. Phenomenological K-like Inhibitory Alternative

Under K-ON conditions, each neuron receives an inhibitory mechanosensitive K^+^ current

(11) $$I_{K,\mathrm{mech}}=g_{K,\mathrm{mech}}\left(\sigma \right)\left(V-E_{K}\right)$$

with $E_{K}=-77$ mV and stress-dependent conductance

(12) $$g_{K,\mathrm{mech}}\left(\sigma \right)=g_{\max,K}\cdot \frac{1}{1+\exp\;\left(-\frac{\sigma -\sigma_{\mathrm{half},K}}{k_{K}}\right)}.$$

K-OFF was represented by $g_{\max,K}=0$. K-ON candidates used

$$\begin{array}{cc} g_{\max,K} & \in \left\{0.1,0.2,0.35,0.5,1.0\right\}\;{\mathrm{mS}/\mathrm{cm}}^{2};\\ \sigma_{\mathrm{half},K}/\sigma_{\mathrm{half},i} & \in \{24,30,36\};\\ k_{K}/\sigma_{\mathrm{half},K} & \in \{0.064,0.080,0.096\}. \end{array}$$

These quantities are phenomenological population-scale parameters. Quantitative TRAAK gating provides the literature background for an outward-current surrogate [20]. However, effective bulk stress is not a molecular membrane-tension threshold, and the branch does not identify a channel.

Within the model, low-intensity conditions generally keep σ below the K^+^ threshold, so the synaptic pathway dominates. At sufficiently high modelled stress, σ can exceed $\sigma_{\mathrm{half},K}$. The outward K^+^ current can then compete with $I_{\mathrm{syn}}$. This fitted inhibitory surrogate tests whether an outward-current form can describe high-intensity response rolloff. Its frequency-dependent contribution is evaluated by the fixed-map ablation analysis.

### 2.9. Shared Observation Model and Nested Leave-One-Frequency-Out Assessment

For frequency *f* and intensity index *i*, $y_{fi}$ denotes the author-provided mean response corresponding to the published three-frequency curves, and ${\mathrm{SEM}}_{fi}$ is its associated standard error. The published response $y_{fi}$ and simulated mean spike count $m_{fi}$ were linked by one affine observation model shared across all frequencies:

(13) $$\mu_{fi}=b+a\;m_{fi},\;a\geq 0.$$

Here, $\mu_{fi}$ is the predicted mean on the published response scale for frequency *f* and intensity point *i*, whereas $m_{fi}$ is the corresponding simulated mean spike count. The parameters *a* and *b* are the cross-frequency shared slope and intercept. The observation variance was

(14) $$V_{fi}={\mathrm{SEM}}_{fi}^{2}+a^{2}{\mathrm{SE}}_{\mathrm{sim},fi}^{2}+\tau^{2}.$$

The Gaussian likelihood includes the published SEM, simulation uncertainty, and a shared residual scale τ. The slope *a*, intercept *b*, and τ were shared by 1.9, 15, and 43 MHz. Frequency-specific rescaling was prohibited. This statistical mapping places the two endpoints on a common response scale; it does not make simulated active-window spike counts biologically equivalent to the published experimental response.

Conditional on the simulated output and observation mapping, we assumed

(15) $$y_{fi}∼N\left(\mu_{fi},V_{fi}\right).$$

For a training set T, this Gaussian observation model gives the negative log likelihood

(16) $${\mathrm{NLL}}_{T}=\frac{1}{2}\sum_{(f,i)\in T}\left[\log\left(2\pi V_{fi}\right)+\frac{{\left(y_{fi}-\mu_{fi}\right)}^{2}}{V_{fi}}\right],\;\mathrm{NLPD}=\frac{\mathrm{NLL}}{n}.$$

Minimising NLL over the training frequencies is equivalent to maximum-likelihood selection under this error model and weights observations by their total uncertainty. The null comparator did not use the simulated output $m_{fi}$; it comprised an intercept and residual variance fitted only to the same training frequencies.

We used a nested leave-one-frequency-out model-selection assessment. Within each fold, all candidate selection and fitting of *a*, *b*, and τ used only the other two frequencies. The fixed candidate library combined 42 shared-threshold profiles, the nine $\left(a_{e},a_{i}\right)$ pairs, three OU amplitudes shared across frequencies, K-OFF, and the K-ON grid defined above. A deterministic block-coordinate search started at the grid centre with K-OFF and updated, in order, the threshold profile, synaptic-amplitude pair, shared OU amplitude, and K branch. Each candidate required a new fit of *a*, *b*, and τ on the training frequencies. The search stopped when a complete round made no change, or after three rounds. Ties favoured the simpler model and then the predefined candidate order.

$Q_{\mathrm{LOFO}}^{2}$ and the root mean square error (RMSE) assessed held-out mean-response prediction, whereas NLPD, standardised residuals, and 95% prediction-interval coverage assessed probabilistic calibration. The training-only null prediction was used in

(17) $$Q_{\mathrm{LOFO}}^{2}=1-\frac{\sum {\left(y-\hat{y}\right)}^{2}}{\sum {\left(y-{\hat{y}}_{\mathrm{null}}\right)}^{2}}.$$

Candidate screening and each held-out fold used separate sets of 10 independent random seeds at N=15 and K=2. These seed sets were mutually disjoint. The final full-data candidate selection repeated the same search on all three frequencies using the screening settings. After selection, the chosen model was re-estimated with 30 independent random seeds, disjoint from all screening and held-out evaluation sets, at N=60 and K=3 to produce the final curves and downstream comparisons. The candidate library was fixed before this reanalysis but was not prospectively preregistered. The nested assessment therefore reduces within-fold selection leakage but is not independent external validation.

### 2.10. K-like Inhibitory-Branch Ablation

After final full-data selection, we compared the selected branch with its K-OFF counterpart at N=60, K=3, and 30 independent random seeds disjoint from the selection folds. Both branches used the same threshold profile and observation mapping, which was not refitted after ablation. The primary measure was the change in negative log predictive density (NLPD), defined as K-OFF minus the selected full-data branch. Positive values favour the selected branch. Random seeds quantify Monte Carlo variation and were not treated as biological replicates for significance testing.

### 2.11. Fixed-Map Mechanism Comparisons

To test whether the descriptive benefit of the high-threshold outward-current branch could instead be reproduced by a simpler direct mechanosensitive excitatory current, we compared both alternatives without allowing either to acquire a new response-scale fit. At N=60, K=3, and the same 30 independent final seeds, we compared the selected full-data branch, K-OFF, and K-OFF plus an incremental direct-current comparator. For the last comparator only, $I_{\mathrm{mech}}=g_{\mathrm{mech},\max}p_{\mathrm{open}}\left(V-E_{\mathrm{mech}}\right)$ with $g_{\mathrm{mech},\max}=0.15$ mS/cm^2^, $E_{\mathrm{mech}}=0$ mV, and $p_{\mathrm{open}}$ using the same relative logistic slope $k_{s,i}=\sigma_{\mathrm{half},i}/2$. The shared synaptic core remained active in this comparator, which therefore did not represent pure direct stimulation; $I_{\mathrm{mech}}$ was zero in every primary-model run. This comparison tested whether a simpler excitatory alternative could reproduce the descriptive contribution of the K^+^ surrogate. All alternatives used the final shared observation mapping without refitting, so relative NLPD measured performance on the same response scale. The deterministic phenomenological comparator was excluded because it does not emit the same raw spike-count endpoint and would require an additional scale parameter.

A supplementary synthetic recovery analysis confirmed the expected score direction for simulated K-ON and K-OFF targets. It was used only to check interpretation of the paired score; detailed settings are provided in the Supplementary Methods.

### 2.12. Monte Carlo Stability Assessment

For each of the 30 final seeds, we applied the fixed full-data observation mapping without refitting. We summarised seed-level NLPD and RMSE and calculated the maximum pointwise standard deviation of the mapped response. These quantities assess numerical stochastic stability, not experimental uncertainty.

### 2.13. Final Full-Data Model and Scope

All primary analyses used one final full-data parameter set selected by the same candidate library and search order used in the nested folds. The OU amplitude was shared across frequencies. Mean-response performance was assessed by $Q_{\mathrm{LOFO}}^{2}$ and the RMSE, while NLPD and interval coverage characterised probabilistic calibration. All quantitative model selection and scoring used the single three-frequency ex vivo retinal dataset of Menz et al. [14]. Nested leave-one-frequency-out assessment therefore tests transfer among frequencies within this dataset, not generalisation across laboratories, species, preparations, or exposure protocols. The full-data calibration and threshold-distribution sensitivity analysis are descriptive fixed-map analyses rather than additional held-out validation. The independent tissue displacement–population association, model comparisons, recovery tests, and exposure-context triage provided complementary checks on interpretation. Alternative thermal, cavitation-related, retinal-network, and mixed-mechanism explanations were not formally compared within this cross-frequency mean-response benchmark.

### 2.14. Illustrative Exposure-Context Triage Calculations

At each experimental $I_{\mathrm{SPPA}}$ point and frequency, we computed three illustrative exposure-context indices. This calculation was designed only to flag exposure conditions requiring dedicated follow-up; it is not a safety assessment. The mechanical index was defined as

(18) $$\mathrm{MI}=\frac{P_{\mathrm{NP}}\;\left[\mathrm{MPa}\right]}{\sqrt{f\;[\mathrm{MHz}]}},$$

where $P_{\mathrm{NP}}=\sqrt{2\rho c\;I_{\mathrm{SPPA}}}$. The spatial-peak temporal-average intensity was estimated as

(19) $$I_{\mathrm{SPTA}}=I_{\mathrm{SPPA}}\times D_{f}$$

for illustrative duty factors $D_{f}=0.01$ and 0.10, representing low and higher repeated-exposure scenarios rather than a reconstruction of a specific experimental protocol. A specified-assumption adiabatic temperature estimate was calculated as

(20) $$\Delta T=\frac{2\alpha \left(f\right)\cdot I_{\mathrm{SPPA}}\cdot t_{\mathrm{pulse}}}{\rho \cdot C_{p}},$$

with $t_{\mathrm{pulse}}=0.1$ s, ρ=1050 kg/m^3^, and $C_{p}=3600$ J/(kg·K). The assumed duty factors and pulse duration do not reconstruct the original protocols. The calculation also omits a measured three-dimensional ocular field, realistic eye geometry and boundaries, diffusion, perfusion, repeated-pulse heat accumulation, and direct temperature measurements. Accordingly, diagnostic-ultrasound reference documents are cited only as contextual background and cannot be converted into a safety or practical-feasibility determination for retinal stimulation [32,33,34]. Complete assumptions are detailed in the Supplementary Methods.

## 3. Results

### 3.1. Tissue Displacement–Population Response Association

**Figure 2:**
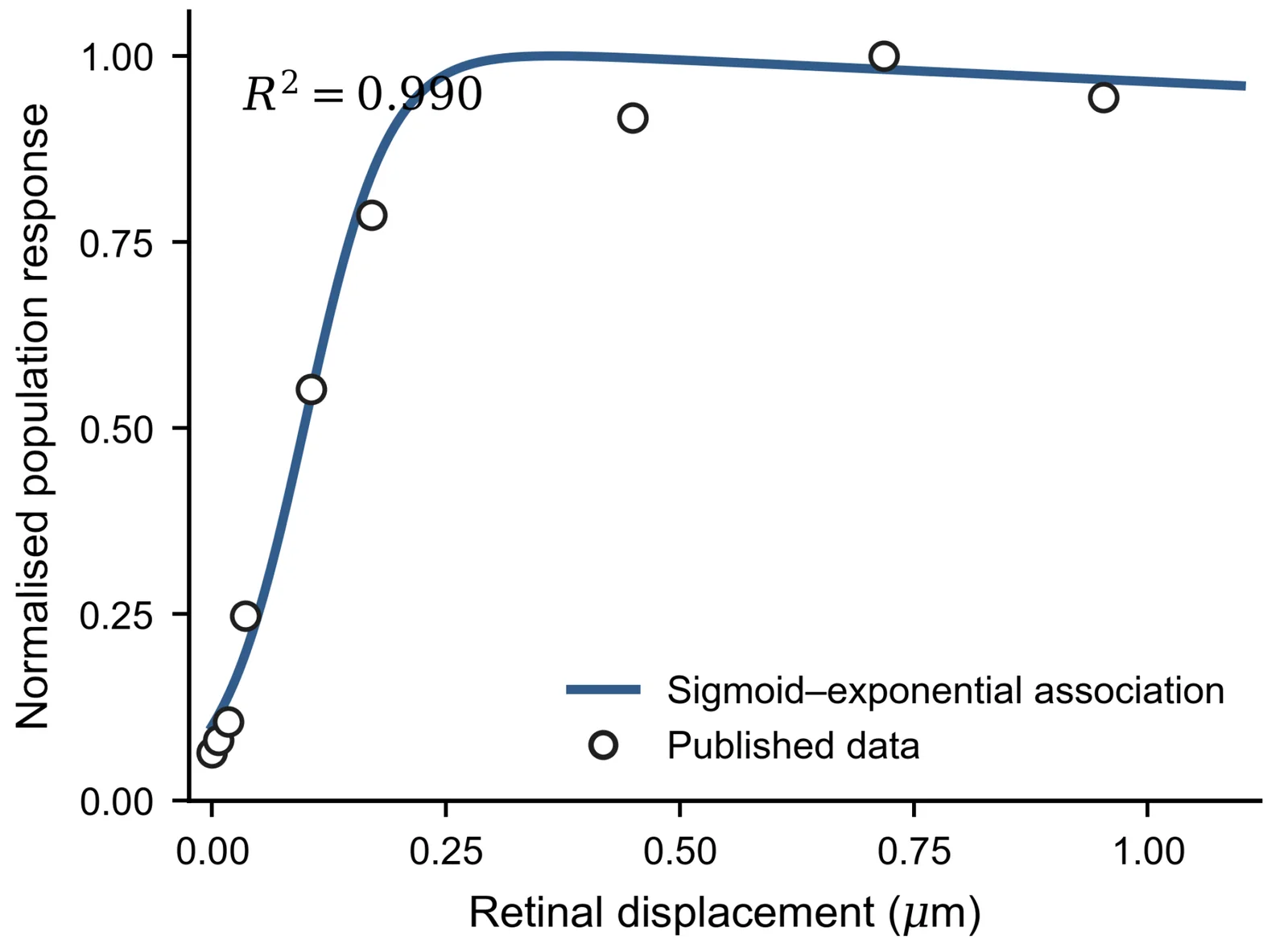
Independent tissue-level association fit to the published tissue displacement–population response data from Menz et al. [14] (n=9 points). Open circles show the published observations. The solid curve shows the fitted sigmoid-times-inhibitory-exponential association ($R^{2}=0.990$, $x_{0}=0.100\;\mu$m, $k_{0}=22.3$ μm^−1^, and $x_{\mathrm{inh}}=16.8\;\mu$m).

The fitted curve closely described the nine published observations. Its approximately 100 nm midpoint summarises a tissue-level association between separate measurements; it is not a membrane threshold, paired single-cell measurement, or HH calibration parameter (Figure 2).

### 3.2. Nested Leave-One-Frequency-Out Assessment on a Shared Response Scale

**Figure 3:**
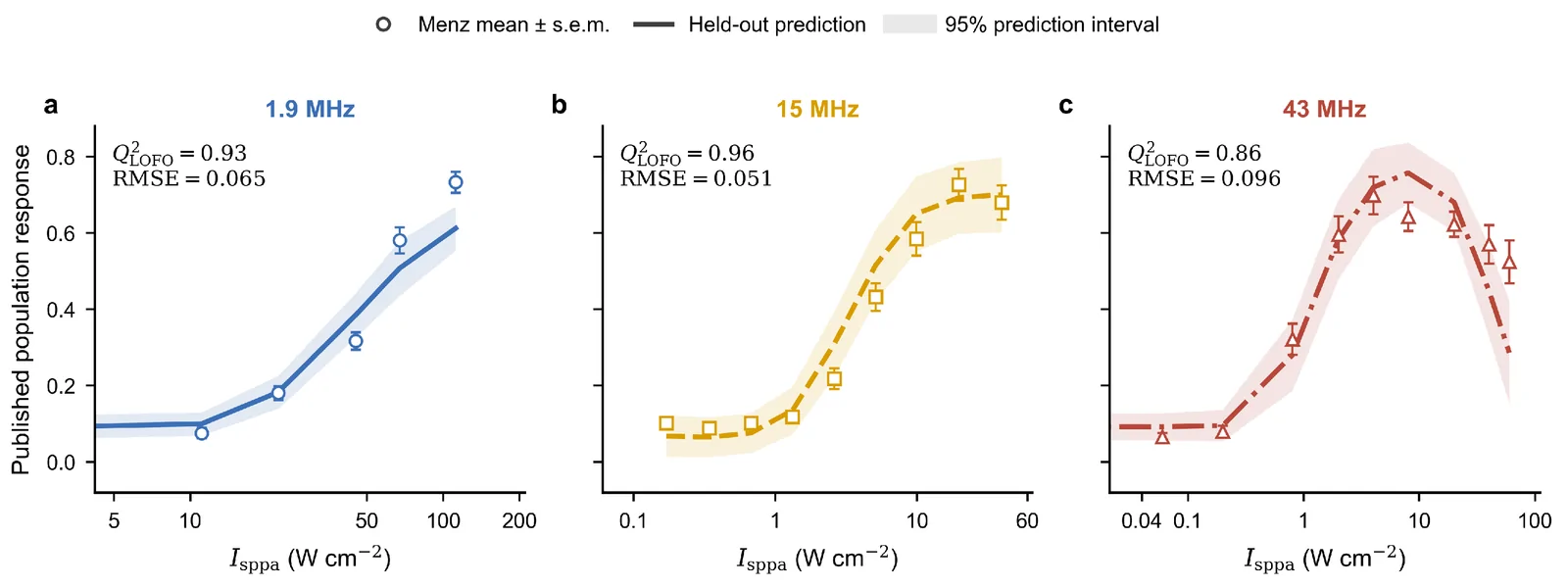
Nested leave-one-frequency-out assessment on the published response scale. (**a**) Held-out predictions at 1.9 MHz. (**b**) Held-out predictions at 15 MHz. (**c**) Held-out predictions at 43 MHz. Each panel shows one frequency excluded from all candidate selection and observation-model fitting in that fold. Symbols and error bars are published mean ± SEM values. Lines and shaded bands show held-out predictions and 95% prediction intervals derived from the shared observation model and its variance components; they are not intervals for biological replicates. No frequency-specific response rescaling was used. Panel annotations report $Q_{\mathrm{LOFO}}^{2}$ and the RMSE; complete probabilistic diagnostics are reported in Supplementary Table S3.

Across 25 held-out points, the model achieved an overall $Q_{\mathrm{LOFO}}^{2}=0.915$ and RMSE of 0.0752. Mean-response prediction was most accurate at 15 MHz ($Q_{\mathrm{LOFO}}^{2}=0.960$, RMSE 0.0514), followed by 1.9 MHz ($Q_{\mathrm{LOFO}}^{2}=0.934$, RMSE 0.0649) and 43 MHz ($Q_{\mathrm{LOFO}}^{2}=0.865$, RMSE 0.0961). Overall NLPD was −0.970, compared with 0.063 for the training-only null model. Predictive-density gains were largest at 15 and 43 MHz and smaller at 1.9 MHz, whereas uncertainty calibration was weakest at the lowest frequency. Complete fold-specific probabilistic diagnostics are reported in Supplementary Table S3. The held-out predictions are shown in Figure 3.

The final full-data calibration selected $\mu_{\log}=-0.25$ and $\sigma_{\log}=0.60$. The shared mapping had a=0.0798, b=0.0462, and τ=0.0278. This calibration is descriptive because all three frequencies contributed to selection and fitting. Supplementary Figure S1 reports the descriptive fitted curves on the shared response scale.

### 3.3. Threshold-Distribution Sensitivity Under the Fixed Mapping

The moment-matched log-normal, Gamma, and Weibull threshold families produced overall RMSEs of 0.0434, 0.0445, and 0.0461 and overall NLPDs of −1.7480, −1.6879, and −1.6233, respectively. Their 95% interval coverage was 1.000, 1.000, and 0.667 at 43, 15, and 1.9 MHz for every family. The fixed-map K-OFF minus K-ON NLPD difference remained positive overall for all distributions (0.2194, 0.2376, and 0.2353; positive in 100% of 2000 paired bootstrap resamples) and was concentrated at 43 MHz; the frequency-specific K-ablation differences at 15 and 1.9 MHz were near zero or slightly negative (Table S4). Thus, the main qualitative ablation conclusion persisted across the three positive-valued threshold families, although mean-response and lower-frequency predictive-density metrics were not identical. Complete frequency-specific results and bootstrap intervals are reported in Supplementary Table S4.

### 3.4. Monte Carlo Stability on the Shared Response Scale

**Figure 4:**
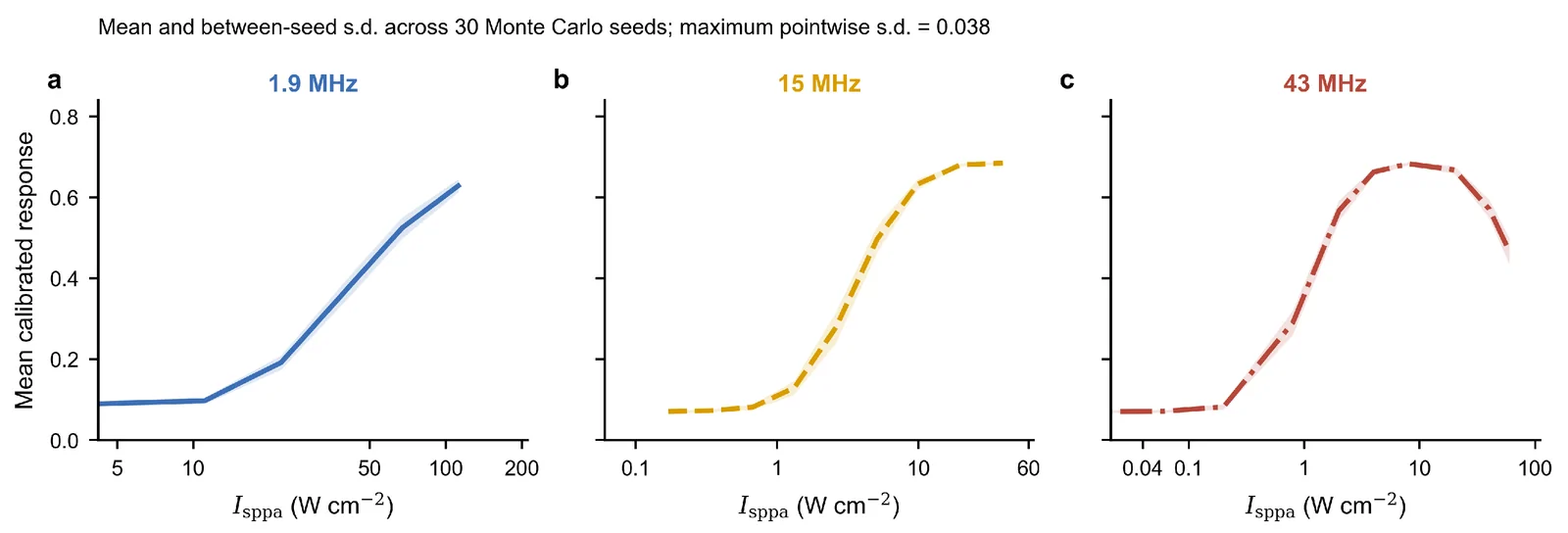
Monte Carlo stability under the fixed shared observation model. (**a**) Across-seed response profile at 1.9 MHz. (**b**) Across-seed response profile at 15 MHz. (**c**) Across-seed response profile at 43 MHz. Curves show mapped mean responses and across-seed SD for 30 computational realisations. The seeds quantify numerical stochastic variation and are not biological replicates.

Across 30 final seeds, the mean NLPD was −1.659 (SD 0.140) and the mean RMSE was 0.0467 (SD 0.0053). The largest pointwise standard deviation on the mapped response scale was 0.0379. The observation mapping was fixed and was not refitted for individual seeds. The across-seed response profiles are shown in Figure 4.

### 3.5. Exploratory Inhibitory-Branch Ablation

**Figure 5:**
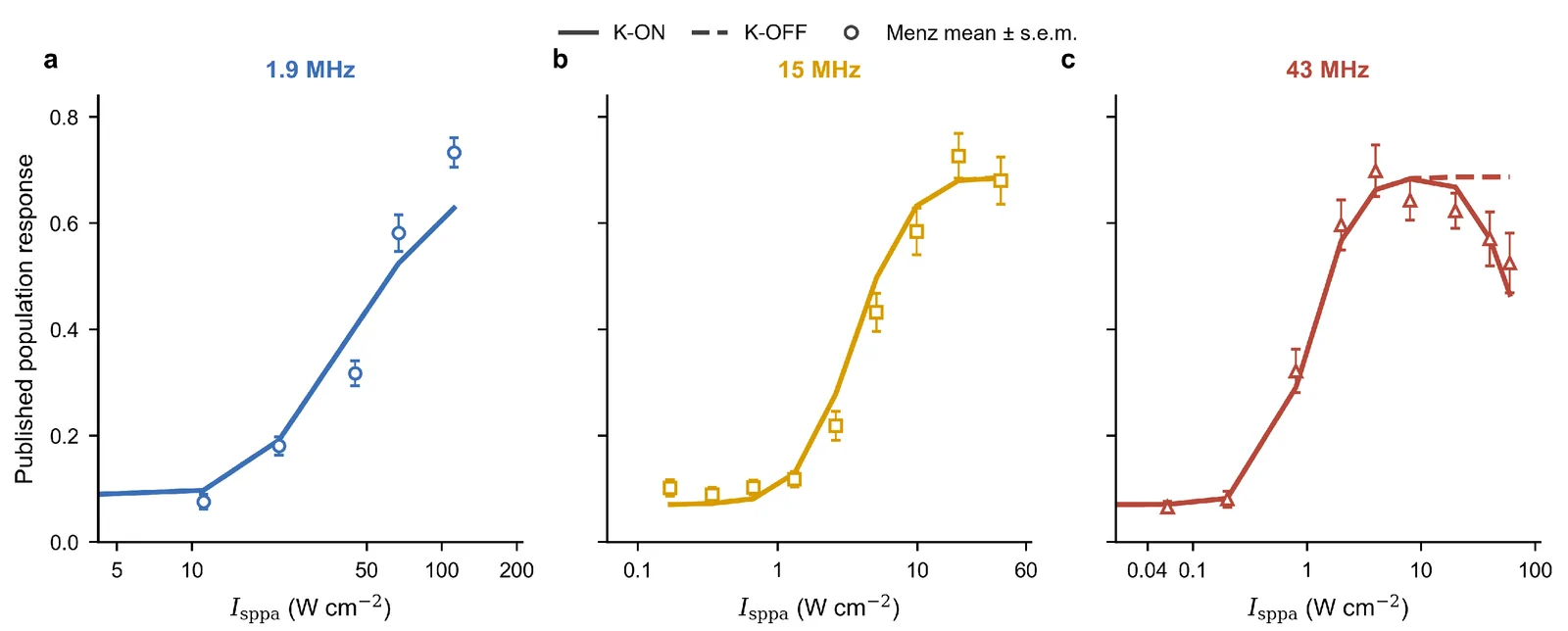
K-like inhibitory-branch ablation under a fixed observation model. (**a**–**c**) Published responses and mapped K-ON and K-OFF predictions at 1.9, 15, and 43 MHz, respectively. Both branches use the same threshold profile and observation mapping. Random seeds quantify Monte Carlo variation and were not treated as biological replicates.

Under the fixed observation mapping, removing the K-like branch increased the overall NLPD by 0.219. The change arose mainly at 43 MHz (ΔNLPD 0.548); the 15 MHz change was 0.00053 and the 1.9 MHz change was −0.00027. Under the current parameterisation, the branch therefore contributed primarily to the 43 MHz high-intensity rolloff, supporting an outward-current-like functional hypothesis for experimental testing rather than a specific molecular-channel assignment (Figure 5).

### 3.6. Mechanism Comparators Define the Interpretation Boundary

**Figure 6:**
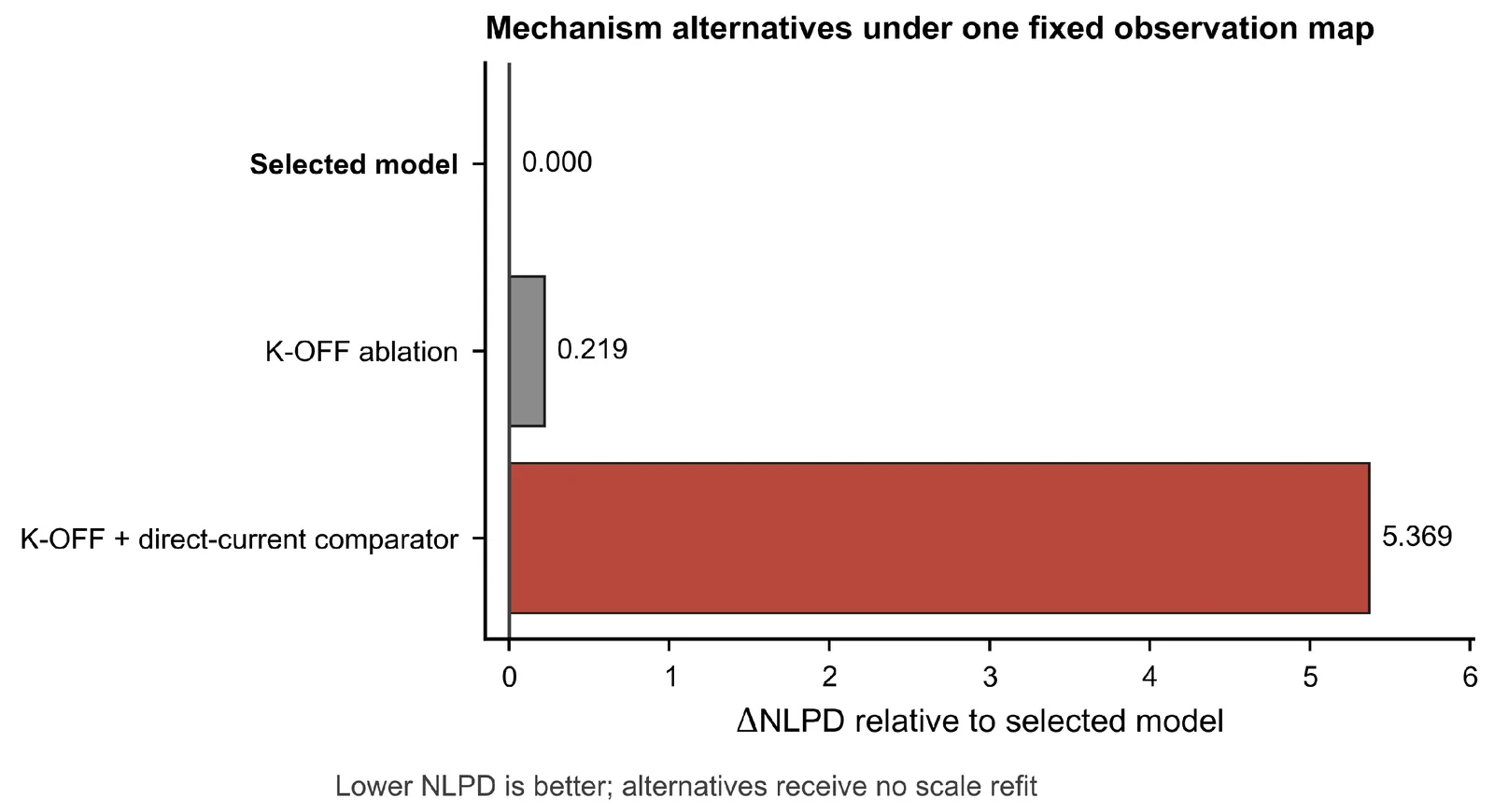
Mechanism alternatives under the fixed shared observation model. Bars show ΔNLPD relative to the selected full-data model. The observation mapping was fixed across alternatives. Positive ΔNLPD indicates worse predictive density for the alternative.

This comparison tested whether the improvement associated with the K-like outward-current branch could be reproduced by adding a simpler direct mechanosensitive excitatory current to the K-OFF model under the same fixed observation mapping (Figure 6).

K-OFF increased NLPD by 0.219 relative to K-ON. Adding the direct-current comparator to K-OFF increased NLPD by 5.369 relative to K-ON. This comparison applies only to the specified fixed direct-current form, which was not re-optimised. It does not exclude other direct mechanosensitive or mixed pathways. The deterministic phenomenological comparator was not scored because it lacks the same raw spike-count endpoint and would require another scale parameter.

### 3.7. Illustrative Exposure-Context Triage

The calculated indices varied with peak intensity and the assumed duty factor. At EC_50_, the specified-assumption single-pulse adiabatic temperature estimates remained below 1 ∘C at all three frequencies. At the highest tested intensities, they exceeded 1 ∘C at 15 and 43 MHz, and several hypothetical temporal-average comparisons exceeded the cited ophthalmic diagnostic reference, particularly at higher intensities or assumed duty factors. These observations only flag conditions that would require dedicated protocol-specific acoustic and bioheat analysis. Supplementary Figure S2 shows the complete triage.

## 4. Discussion

This study establishes a practical computational route from ultrasound exposure to cross-frequency neural response prediction. The route connects an ARF-based tissue-scale input, a heterogeneous stochastic HH population, one shared observation mapping, and nested leave-one-frequency-out assessment. Its central contribution is to place the physical input, population response, and alternative mechanism branches on one response scale without frequency-specific rescaling. Within this structure, the model retained the principal mean-response pattern of the published three-frequency RGC data. Because each held-out frequency was excluded from fold-specific candidate selection and mapping, the analysis tests internal transfer across frequencies within that dataset; it is not an independent validation across laboratories, species, preparations, or protocols.

The shared observation mapping is the key methodological constraint in this route. Separate normalisation at each frequency would require the model mainly to reproduce within-frequency curve shapes. Here, one slope, intercept, and residual scale mapped all unnormalised simulated spike counts to the published response scale. The model therefore had to preserve the coupled effects of ARF scaling, population-threshold crossing, and excitable-membrane output across carrier frequencies. This provides a more demanding and reusable basis for comparing physical and biophysical hypotheses than frequency-by-frequency curve fitting.

Mean-response prediction and probabilistic calibration provided complementary information. The $Q_{\mathrm{LOFO}}^{2}$ and RMSE values show that the mean response trend at 1.9 MHz was captured, but the less favourable NLPD and the nominal 95% prediction interval covering only 50% (0.50) of held-out points identify the reliability of its predictive distribution as weaker than at 15 and 43 MHz. Under the adopted attenuation law, the effective stress at 1.9 MHz is smaller, increasing the influence of the low-threshold population tail, OU fluctuations, and the shared residual term. Acoustic-field scale, bulk tissue motion, boundary reflections, streaming, or additional transduction processes may also become relatively more important. Pressure-field and standing-wave effects are known to shape ultrasonic neuromodulation in other preparations [35], but the present data cannot assign a specific cause. Measurements at low and intermediate frequencies could distinguish these possibilities and directly test where the present ARF proxy requires refinement. These results make 1.9 MHz a priority regime for model refinement rather than an equally well-calibrated part of the benchmark.

The phenomenological K-like inhibitory branch adds a second experimentally accessible prediction. Its fixed-map ablation shows that a high-threshold inhibitory mathematical form improves description of the high-intensity rolloff, mainly at 43 MHz; it does not localise that inhibition to an RGC or identify a potassium channel. In the model, increasing stress first strengthens synaptic drive and then recruits a threshold-dependent outward current that opposes depolarisation, producing a non-monotonic response. This predicts that perturbations of outward K^+^ drive should preferentially alter the high-intensity downturn while preserving more of the lower-intensity rising phase. Mechanosensitive K2P channels such as TREK/TRAAK provide illustrative biophysical context only [19,20], without implying that the fitted branch corresponds to that channel family. Alternative explanations include GABAergic or glycinergic inhibition delivered by retinal interneuron circuits [36], or depletion/adaptation of excitatory synaptic drive at high intensity. The present ablation cannot distinguish these cellular, circuit, and synaptic sources; it provides a testable functional hypothesis rather than evidence for molecular specificity.

A broader contribution of the workflow is that alternative explanations can be challenged under the same observation mapping and predictive scoring framework. The fixed direct-current comparator performed substantially worse than the selected model, indicating that monotonic mechanosensitive excitation did not reproduce the high-intensity rolloff captured by the outward-current branch. Thermal effects, acoustic-field structure, network inhibition, synaptic depletion, and mixed mechanisms remain experimentally relevant alternatives, but they can now be introduced as explicit model branches and evaluated without changing the response scale after the fact. The framework therefore provides a common quantitative testbed for moving from plausible mechanisms to discriminating predictions. The candidate mechanisms and their status in the present study are summarised in Table 1.

The threshold-family analysis provides an additional structural check. Matching the mean and coefficient of variation prevented scale differences from dominating the comparison, and common quantiles paired the same neuron ranks across distributions. Log-normal, Gamma, and Weibull thresholds yielded modestly different RMSE and NLPD values, especially at 1.9 MHz, but all preserved the positive overall K-ablation score and its concentration at 43 MHz. The conclusions are therefore qualitatively stable across these three positive-valued families, not invariant to the distributional assumption. Cell-resolved mechanical thresholds were not available, so no tested family should be interpreted as a measured RGC threshold distribution.

The scope of the current implementation is set by three main approximations. The affine observation mapping links simulated spike counts to a published population-response quantity; the standard HH population represents excitable-membrane dynamics without resolving RGC subtypes; and the effective bulk-stress proxy compresses spatially structured retinal mechanics into one input variable. Together with the three available carrier frequencies, these choices define the present analysis as a population-level, shared-scale assessment. The exposure-context calculation similarly identifies conditions requiring additional thermal evaluation, while protocol-specific feasibility will depend on pulse timing, heat transport, and the acoustic field.

Lu et al. reported frequency-dependent ultrasonic retinal stimulation in a different experimental setting, providing qualitative external context for a frequency–threshold relation [44]. Differences in species, endpoints, acoustic fields, and protocols preclude pooling or treating that study as quantitative validation. Single-cell profiling has identified extensive molecular diversity among RGC types [45]. Effective threshold heterogeneity can absorb only part of this unresolved diversity and does not replace an explicit retinal circuit model. Future versions can replace the shared HH component with subtype-specific conductance models and explicit retinal circuits while retaining the shared-scale evaluation principle.

The exposure-context calculation is illustrative triage rather than a safety assessment. Its assumed duty factors and adiabatic pulse estimate omit protocol-specific three-dimensional acoustics, realistic ocular boundaries, heat diffusion and perfusion, repeated-pulse accumulation, and measured intraocular temperature. Direct experiments show that ultrasound can heat ocular tissues under some exposure conditions [34]; diagnostic guidance documents [32,33] provide background reference values only. A defensible safety evaluation would require protocol-specific three-dimensional acoustic and bioheat modelling, direct temperature measurements, retinal function and tissue-injury endpoints, and repeated-exposure assessment. No conclusion about ocular safety or practical feasibility is drawn here.

These approximations also define a clear route for extension. Prospective measurements at additional carrier frequencies can test the ARF exponent and population-threshold distribution. Paired displacement or strain measurements and retinal electrophysiology can connect the mechanical and neural stages more directly. Perturbations of K^+^ driving force, network inhibition, and temperature can separate outward-current, circuit-level, and thermal contributions. Because the acoustic, mechanical, neuronal, and observation modules are explicit and separable, spatially resolved fields or cell-type-specific models can replace the current reduced components without changing the shared-scale evaluation principle. Taken together, this work provides a stepwise route for converting isolated frequency-dependent dose–response curves into cumulative, testable models of ultrasound neuromodulation.

A direct test of the predicted rolloff should combine ex vivo retinal multi-electrode array or intracellular recordings with tissue displacement/local strain and temperature measurements. At 43 MHz, intensities should span the rising phase, the response maximum, and the downturn, with 1.9 and 15 MHz controls. Two independent perturbation routes should be used: one altering outward K^+^ driving force or conductance, and another separately altering GABAergic and glycinergic inhibition. A predominantly cell-autonomous outward-current mechanism predicts a selective change in the high-intensity downturn with less effect on the low-intensity rise; a circuit mechanism predicts a larger rolloff change after inhibitory synaptic perturbation. Covariation of the downturn with temperature or field-dependent tissue motion would instead require thermal or acoustic-structure explanations. These tests should be specified prospectively and do not presume that any single pharmacological intervention is uniquely selective.

## 5. Conclusions

An ARF-centred HH population model with one shared observation mapping retained the main held-out mean-response structure of the published three-frequency data set. By applying one response scale across frequencies, the framework evaluates frequency-dependent ARF input, population threshold heterogeneity, and nonlinear membrane excitability without frequency-specific response rescaling. Moment-matched log-normal, Gamma, and Weibull thresholds produced modest score differences but preserved the K-like improvement, mainly at 43 MHz. The K-like inhibitory branch further generates a testable prediction: a high-threshold inhibitory functional process improves the description of high-intensity rolloff without identifying a specific molecular channel or cellular source.

Fixed-map prediction, Monte Carlo analysis, and mechanism ablation together provide a modular route for testing how acoustic exposure may propagate through tissue-scale mechanics to population firing. The current evidence supports this route as a cross-frequency mean-response benchmark, while molecular identity and ocular safety require separate experimental evidence. Additional frequencies and paired mechanical–electrophysiological measurements can extend the framework from retrospective benchmarking towards prospective testing.

## Figures and Tables

**Table 1:** Conceptual comparison of candidate mechanisms and their status in the present study. The table separates quantitative implementation from mechanisms that remain experimental alternatives; it is not a ranking of biological evidence.

Candidate Mechanism	Proximal Driver	Expected Dose–Response Signature	Status in the Present Study	Discriminating Measurement or Perturbation
ARF/shared-scale primary model	Absorption-related body force represented by effective bulk stress [14,25,37]	Frequency-dependent threshold crossing and saturating population excitation	Implemented and quantitatively scored by nested LOFO and fixed-map analyses	Three-dimensional pressure/force reconstruction with paired retinal displacement or strain and electrophysiology
Cavitation/NICE–SONIC	Pressure-driven intramembrane oscillation or cavitation dynamics [15,16,17]	Excitation governed by pressure, carrier frequency, membrane properties, and pulse timing	Discussed but not formally compared; unresolved experimentally in this MHz retinal setting	Cavitation-sensitive acoustic measurements and predictions from a geometry- and frequency-matched model
Thermal contribution	Absorbed energy and temperature change [34,38]	Response associated with pulse duration, duty cycle, cumulative energy, and local temperature	Discussed; only illustrative exposure-context triage, not a mechanistic fit or safety assessment	Direct intraocular temperature measurements and temperature-matched controls
K-like outward-current branch	High-threshold inhibitory functional conductance, with mechanosensitive K2P channels as biophysical context [19,20,22,39]	Selective attenuation of the high-intensity response after an initial rising phase	Implemented and quantitatively scored by fixed-map ablation; molecular and cellular sources unresolved	Intracellular current recording and perturbation of outward-current driving force or conductance
Retinal network inhibition or synaptic depletion	GABAergic/glycinergic interneuron input, contrast-gain control, or adaptation of excitatory transmission [36,40,41]	High-intensity rolloff without requiring a cell-autonomous outward channel	Discussed but not formally compared; unresolved experimentally	Separate perturbation of GABAergic/glycinergic transmission and measurement of excitatory and inhibitory synaptic currents
Direct mechanosensitive excitation	Mechanically gated inward current or excitatory conductance [21,22,42,43]	Predominantly monotonic excitation for the restricted implemented form	Implemented only as a restricted fixed-parameter comparator; other direct or mixed forms are not excluded	Membrane-current recordings with mechanical and synaptic perturbations under a fixed acoustic field

## Data Availability

The processed data, point-level simulation outputs, metadata, and verification code supporting the findings of this study are openly available in the GitHub repository “ARF–RGC Predictive Benchmark” at https://github.com/BingaoZhang927/arf-rgc-predictive-benchmark (accessed on 23 August 2026). The repository contains the materials necessary to reproduce the principal statistical analyses and verify the reported numerical results.

## Supplementary Materials

The following supporting information can be downloaded at: https://www.mdpi.com/article/10.3390/bioengineering13090979/s1, Figure S1: Full-data calibration on the shared response scale; Figure S2: Illustrative exposure-context triage at EC_50_ and the maximum tested intensity; Table S1: Acoustic and tissue parameters; Table S2: Hodgkin–Huxley model parameters; Table S3: Complete nested leave-one-frequency-out diagnostics; Table S4: Fixed-map threshold-distribution sensitivity.

## Abbreviations

The following abbreviations are used in this manuscript:

AMD	Age-related macular degeneration
ARF	Acoustic radiation force
EC_50_	Half-maximal effective intensity
HH	Hodgkin–Huxley
K2P	Two-pore domain potassium
MI	Mechanical index
NICE	Neuronal intramembrane cavitation excitation
OU	Ornstein–Uhlenbeck
NLPD	Negative log predictive density
RGC	Retinal ganglion cell
RMSE	Root mean square error
RP	Retinitis pigmentosa
SD	Standard deviation
SONIC	Submaximal oscillation neuronal intracellular cavitation
SPPA	Spatial-peak pulse-average
SPTA	Spatial-peak temporal-average
TREK	TWIK-related K^+^ channel
TRAAK	TWIK-related arachidonic acid-stimulated K^+^ channel
