# Supplementary material for: Shared-Scale Predictive Benchmarking of an Acoustic-Radiation-Force Model for Frequency-Dependent Retinal Ganglion Cell Responses
Source: Bioengineering (Basel). 2026 Aug 26;13(9):979. doi: 10.3390/bioengineering13090979 (PMC13603775; doi:10.3390/bioengineering13090979)
Supplement: Supplementary file 1 [file bioengineering-13-00979-s001.zip › bioengineering-4511129-supplementary.pdf]

Supplementary Materials: Shared-Scale Predictive Benchmarking of an Acoustic-Radiation-Force Model for Frequency-Dependent Retinal Ganglion Cell Responses

Bingao Zhang and Shengyong Xu *

Department of Electronics, Peking University, Beijing 100871, China.
* Correspondence: xusy@pku.edu.cn

Supplementary Methods

Nested model selection. The candidate library comprised a fixed 7×6 grid of shared log-normal threshold profiles, the synaptic-amplitude combinations and shared OU amplitudes specified in the main Methods, and the absence or presence of an optional phenomenological K-like inhibitory branch (K-OFF/K-ON). The K-ON label denotes a fitted outward-current surrogate, not K2P/TREK/TRAAK channel identity. Within each held-out-frequency fold, all candidate selection and observation-model fitting used only the two training frequencies. The library was fixed before this reanalysis but was not prospectively preregistered; accordingly, this procedure is a nested model-selection assessment rather than independent external validation. Candidate screening and each held-out fold used mutually disjoint sets of 10 independent random seeds at $N = 15$ and $K = 2$. The final full-data analysis used a separate set of 30 independent random seeds at $N = 60$ and $K = 3$.

Numerical implementation checks. We assessed whether the integration step and OU input discretisation affected the reported dose–response trends. Representative simulations were repeated with timesteps of 0.04, 0.02, 0.01, and 0.005 ms and with an independent reference implementation. The empirical mean, stationary variance, and autocorrelation of the OU input were compared with their analytical stationary values. These checks supported the use of $\Delta t = 0.02$ ms for the reported simulations.

Threshold-distribution sensitivity. The selected log-normal profile had $\mu_{\log} = -0.25$ and $\sigma_{\log} = 0.60$, corresponding to mean $\exp(\mu_{\log} + \sigma^2_{\log}/2) = 0.9324$ and coefficient of variation $\sqrt{\exp(\sigma^2_{\log}) - 1} = 0.6583$. The moment-matched Gamma distribution used shape 2.3077 and scale 0.4040. The moment-matched Weibull distribution used shape 1.5513 and scale 1.0368, with the shape obtained numerically from the target coefficient of variation. For each seed, the historical normal variates were retained for the log-normal draw and converted to common uniform quantiles for Gamma and Weibull inverse-CDF sampling; quantiles were clipped to $[10^{-9}, 1 - 10^{-9}]$. All three distributions used the same 30 final seeds (242–271), $N = 60$, $K = 3$, synaptic amplitudes, shared OU amplitude, K-like parameters, and affine observation mapping. The mapping and all model parameters were fixed rather than refitted. Metrics were computed from the across-seed predictive mean and variance. Paired seed-bootstrap intervals used 2000 resamples and maintained pairing across distributions and K-ON/K-OFF runs.

Illustrative exposure-context assumptions. Mechanical index was compared with the general diagnostic reference value 1.9 and the ophthalmic diagnostic reference near 0.23. Hypothetical 1% and 10% duty-factor I_{SPTA} values were compared with general and ophthalmic diagnostic reference levels of 720 and 50 mW/cm^2, respectively [2,3]. These regulatory documents concern diagnostic ultrasound and are used only for background context; they are not acceptance criteria for the present stimulation protocol. The adiabatic estimate used an assumed 0.1-s pulse, $\rho = 1050$ kg/m^3, and $C_p = 3600$ J/(kg·K), and omitted thermal diffusion and perfusion. The three-dimensional acoustic field, eye geometry,

material boundaries, repetition timing, temperature, and cumulative exposure were not reconstructed or measured. The calculations are illustrative triage, not guaranteed bounds, regulatory clearance, a safety assessment, or evidence of practical feasibility [4].

Table S1. Acoustic and tissue parameters.

Parameter	Symbol	Value	Source
Wave speed	c	1540 m/s	[1]
Tissue density	ρ	1050 kg/m^3	Assumed soft-tissue value
Attenuation prefactor	α_0	22.02 dB/cm at 43 MHz	de Korte/Menz-based model parameter
Attenuation exponent	n	1.18	de Korte/Menz-based model parameter
Retinal thickness	L	150 μm	Model scale parameter

Table S2. Hodgkin–Huxley model parameters.

Parameter	Symbol	Value
Membrane capacitance	C_m	1.0 μF/cm^2
Max. Na$^+$ conductance	g_{Na}	120 mS/cm^2
Max. K$^+$ conductance	g_{K}	36 mS/cm^2
Leak conductance	g_{L}	0.3 mS/cm^2
Na$^+$ reversal potential	E_{Na}	+50 mV
K$^+$ reversal potential	E_{K}	−77 mV
Leak reversal potential	E_{L}	−54.4 mV
Resting potential	V_0	−65.0 mV
Spike threshold	V_{thresh}	0 mV
Absolute refractory period	t_{ref}	1.2 ms
Bias current	I_{bias}	3.5 μA/cm^2
Integration timestep	Δt	0.02 ms

Table S3. Complete nested leave-one-frequency-out diagnostics. Q^2_{LOFO} and RMSE assess held-out mean-response prediction; NLPD, ΔNLPD, and 95% interval coverage assess probabilistic calibration. Negative ΔNLPD values favour the ARF–HH model over the training-only null.

Held-out frequency	NLPD	ΔNLPD	Q^2_{LOFO}	RMSE	95% coverage
43 MHz	−0.722	−0.801	0.865	0.0961	0.70
15 MHz	−1.613	−1.671	0.960	0.0514	0.89
1.9 MHz	−0.419	−0.463	0.934	0.0649	0.50
Overall	−0.970	−1.033	0.915	0.0752	—

Table S4. Fixed-map threshold-distribution sensitivity. Gamma and Weibull thresholds were matched to the selected log-normal mean and coefficient of variation. Values in brackets are 95% paired seed-bootstrap intervals based on 2000 resamples. ΔNLPD is K-OFF minus K-ON, so positive values favour the K-like branch. The overall ΔNLPD was positive in 100% of bootstrap resamples for all three distributions.

Panel A. Overall paired-seed metrics

Threshold family	RMSE [95% CI]	NLPD [95% CI]	ΔNLPD [95% CI]
Log-normal	0.0434 [0.0417, 0.0455]	−1.7480 [−1.7880, −1.6957]	0.2194 [0.2120, 0.2254]
Gamma	0.0445 [0.0426, 0.0469]	−1.6879 [−1.7368, −1.6205]	0.2376 [0.2315, 0.2422]
Weibull	0.0461 [0.0440, 0.0487]	−1.6233 [−1.6827, −1.5462]	0.2353 [0.2277, 0.2417]

Panel B. Frequency-specific metrics

Threshold family	Frequency	RMSE	NLPD	95% coverage	ΔNLPD
Log-normal	43 MHz	0.0319	−2.0009	1.000	0.5481
	15 MHz	0.0395	−1.8769	1.000	0.0005
	1.9 MHz	0.0616	−1.1329	0.667	−0.0003
Gamma	43 MHz	0.0288	−2.0335	1.000	0.6015
	15 MHz	0.0406	−1.8227	1.000	−0.0049
	1.9 MHz	0.0662	−0.9098	0.667	−0.0050
Weibull	43 MHz	0.0288	−2.0241	1.000	0.6204
	15 MHz	0.0417	−1.7772	1.000	−0.0127
	1.9 MHz	0.0698	−0.7245	0.667	−0.0344

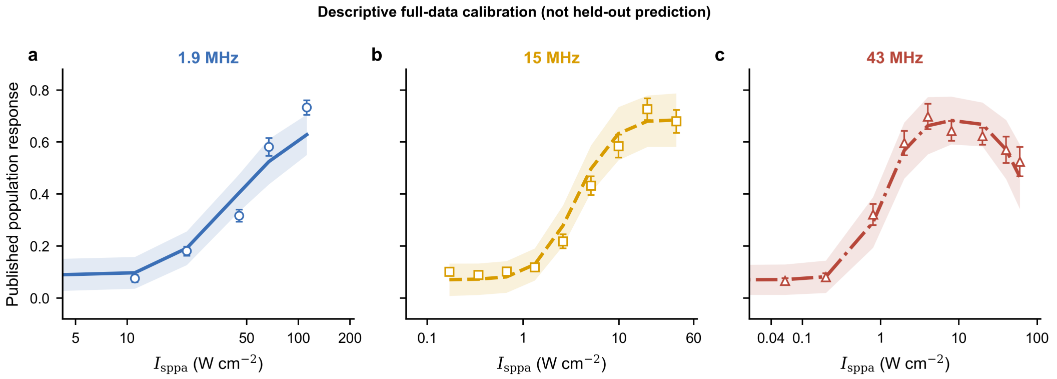

Figure S1. Full-data calibration on the shared response scale. All three frequencies contributed to threshold-profile selection and observation-model fitting, so these curves are retained only as a descriptive calibration view and are not used as held-out predictive evidence.

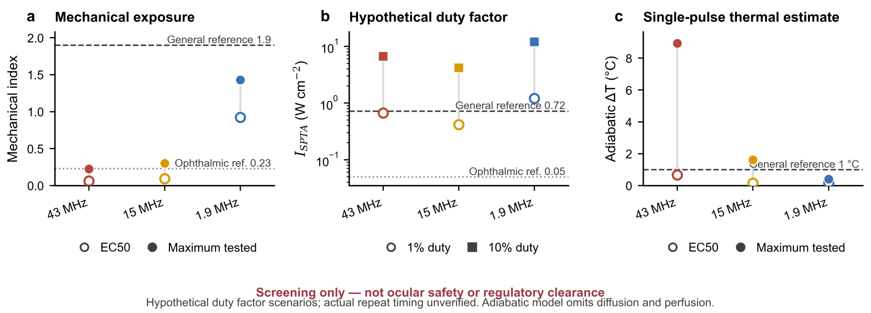

Figure S2. Illustrative exposure-context triage at EC_{50} (open symbols) and the maximum tested intensity (filled symbols). (**a**) Mechanical index with general diagnostic (1.9) and ophthalmic (0.23) references. (**b**) Hypothetical 1% and 10% duty-factor I_{SPTA} scenarios with general (0.72 W cm^{-2}) and ophthalmic (0.05 W cm^{-2}) references. (**c**) Single-pulse adiabatic temperature-rise estimates under the stated assumptions, with a 1°C screening line. Ophthalmic reference levels provide context; actual repeat timing was not verified, and the adiabatic model omits thermal diffusion and perfusion. The calculations are not a protocol-specific safety assessment and do not support conclusions about practical feasibility.

References

1. Menz, M.D.; Ye, P.; Firouzi, K.; Nikoozadeh, A.; Pauly, K.B.; Khuri-Yakub, P.; Baccus, S.A. Radiation force as a physical mechanism for ultrasonic neurostimulation of the ex vivo retina. *J. Neurosci.* **2019**, *39*, 6251–6264. https://doi.org/10.1523/JNEUROSCI.2394-18.2019
2. U.S. Food and Drug Administration. Marketing Clearance of Diagnostic Ultrasound Systems and Transducers: Guidance for Industry and Food and Drug Administration Staff. Final Guidance, February 2023. Available online: https://www.fda.gov/regulatory-information/search-fda-guidance-documents/marketing-clearance-diagnostic-ultrasound-systems-and-transducers (accessed on 18 August 2026).
3. Health Canada. Device licence applications for diagnostic ultrasound systems and transducers: Notice to industry. Health Canada, Ottawa, ON, Canada, 2026. Available online: https://www.canada.ca/en/health-canada/services/drugs-health-products/medical-devices/application-information/guidance-documents/device-licence-applications-diagnostic-ultrasound-systems-transducers-notice-industry.html (accessed on 18 August 2026).
4. King, R.L.; Liu, Y.; Harris, G.R. Quantification of temperature rise within the lens of the porcine eye caused by ultrasound insonation. *Ultrasound Med. Biol.* **2017**, *43*, 476–481. https://doi.org/10.1016/j.ultrasmedbio.2016.09.021
